# Light Quality Dependent Changes in Morphology, Antioxidant Capacity, and Volatile Production in Sweet Basil (*Ocimum basilicum*)

**DOI:** 10.3389/fpls.2016.01328

**Published:** 2016-09-01

**Authors:** Sofia D. Carvalho, Michael L. Schwieterman, Carolina E. Abrahan, Thomas A. Colquhoun, Kevin M. Folta

**Affiliations:** ^1^Horticultural Sciences Department, University of FloridaGainesville, FL, USA; ^2^Environmental Horticulture Department, University of FloridaGainesville, FL, USA; ^3^Plant Innovation Center, Institute of Food and Agricultural Sciences, University of FloridaGainesville, FL, USA; ^4^Plant Molecular and Cellular Biology Program, University of FloridaGainesville, FL, USA

**Keywords:** flavor, aroma, metabolism, nutrition, crop improvement

## Abstract

Narrow-bandwidth light treatments may be used to manipulate plant growth, development and metabolism. In this report LED-based light treatments were used to affect yield and metabolic content of sweet basil (*Ocimum basilicum* L. cv “Ceasar”) grown in controlled environments. This culinary herb produces an aroma highly appreciated by consumers, primarily composed of terpenes/terpenoids, phenylpropanoids, and fatty-acid- derived volatile molecules. Basil plants were grown under narrow-bandwidth light conditions, and leaf area, height, mass, antioxidant capacity and volatile emissions were measured at various time points. The results indicate reproducible significant differences in specific volatiles, and in biochemical classes of volatiles, compared to greenhouse grown plants. For example, basil plants grown under blue/red/yellow or blue/red/green wavelengths emit higher levels of a subset of monoterpenoid volatiles, while a blue/red/far-red treatment leads to higher levels of most sesquiterpenoid volatile molecules. Specific light treatments increase volatile content, mass, and antioxidant capacity. The results show that narrow-bandwidth illumination can induce discrete suites of volatile classes that affect sensory quality in commercial herbs, and may be a useful tool in improving commercial production.

## Introduction

Plants constantly adapt their growth and physiology to best match ambient conditions. Light modulates plant growth throughout development, with effects on seed germination, seedling establishment, transition to flowering, and adaptation to stress responses (Galvão and Fankhauser, 2015). Plants are able to sense and respond to a broad range of the spectrum, spanning from UV-C (260 nm) to the far-red (720–780 nm) regions. Combinations of wavebands in the incident light mixture effect plant growth, development, metabolism and morphology (Folta and Carvalho, 2015).

Plants possess a set of photoreceptors tuned to sense discrete wavebands within the ambient spectrum. Phytochromes typically sense red and far-red light, cryptochromes are activated by UV-A, blue, and green light, and phototropins respond primarily to blue light (Galvão and Fankhauser, 2015). Sensors exist to respond to UV-B (Jenkins, 2014), and specific roles of green light have been noted (Dhingra et al., 2006; Bouly et al., 2007). Stimulation of light sensors activates downstream pathways that ultimately shape plant growth, development, physiology, metabolism, and behavior. These pathways can act independently or show overlapping interactions, imparting unique, antagonistic or synergetic effects of different light wavelengths on plant biology (Folta and Carvalho, 2015).

Activation of discrete limbs of the light-input network allows control of specific plant traits in horticultural crops (Carvalho and Folta, 2014a). For instance, the shade of leaves lowers the red/far-red ratio, and induces the elongation of stems and leaves with an upward reorientation (Fankhauser and Batschauer, 2016). The enrichment in green wavelengths or inadequate blue light also causes shade symptoms (Vandenbussche et al., 2005; Wang and Folta, 2013). UV or blue-enriched environments can induce the accumulation of anthocyanins and carotenoids (Li and Kubota, 2009). Blue light can control plant stature by limiting stem elongation (Neff and Chory, 1998; Briggs and Huala, 1999) or promoting leaf expansion (Wang et al., 2015). All of these examples show opportunity to control specific processes in plants with application of discrete light qualities. Such manipulations may be accomplished using narrow-bandwidth light, such as that produced by light-emitting diode (LED)-based light sources.

In addition to wavelength-specific effects on morphology, development and growth, fluctuations in the ambient light spectrum can be used to change the sensory quality of fruits and vegetables, primarily by modulating the prevalence of specific volatile metabolites (Loughrin and Kasperbauer, 2003). Light has been shown to affect volatile compounds in petunia flowers, in fruits (such as strawberry, blueberry, and tomato) and in tea leaves (Colquhoun et al., 2013; Fu et al., 2015).

This report details how specific light treatments can affect relevant plant traits, such as aroma, in sweet basil (*Ociumum basilicum*). Basil is a highly valued horticultural crop that presents a complex aroma profile. It stands as an excellent system to test the hypothesis that spectral quality can control the critical sensory attributes of herbs, and generate new questions about how broad suites of volatiles may be affected by particular light qualities.

## Materials and methods

### Plant materials and growth conditions

Seeds from basil (*Ocimum basilicum*) “Ceasar” were sown in soil and allowed to germinate in greenhouse or LED-illuminated chambers. Greenhouse plants were grown for 7 days under intermittent mist, and then transferred to a glass-covered greenhouse. Under LEDs seedlings were covered with a transparent plastic lid for 7 days and then were grown for an additional 2 weeks. After the first week of growth, fertigation was initiated with Peters Professional 15-5-15 Cal-Mag at 150 ppm, and maintained during the entire time of growth. At 4 weeks basil plants were transferred to individual pots.

Quantum energy distribution of full sunlight in the greenhouse and in LED chambers was determined at 1 pm in July at the University of Florida, Gainesville, FL (29.67°N), using a StellarNet spectroradiometer. Fluence rates were measured with a LI-COR light meter (model LI-250), and far-red fluence rates were determined with an International Light meter (model IL1400A). LED chambers were maintained at 24°C, ventilated and lined with reflective mylar. Light was provided by Plant Whisperer light units (Light Emitting Computers, Victoria, BC, Canada). The wavelengths used were 450 (Blue—B), 520 (Green—G), 600 (Yellow/Amber—Y), 600 (Red—R), and 735 (Far-red—Fr) nm. Fluence rates were set at 100 μmol m^−2^ s^−1^ for exclusive B or R and for the sum of B and R (1:1 ratio—50 μmol m^−2^ s^−1^ each). When a third wavelength was added (G, Y, or Fr), its contribution was 50 μmol m^−2^ s^−1^ (ratio 1:1:1), giving a total fluence rate of 150 μmol m^−2^ s^−1^. Photoperiod was 12 h light/12 h dark in order to approximate greenhouse photoperiod conditions.

### Growth and developmental assays

Basil plants germinated in the greenhouse were allowed to develop for 6 weeks. At week 2 and once every week (5 measurements), plants were imaged from above. These images were used for leaf area measurement using Image Tool 3.0. Top leaves and internodes from individual seedlings and plants were used for volatile collection. At weeks 2 and 3 entire seedlings were used, whereas from weeks 4 to 6, plants were cut 1 cm below the top internode with a razor blade. This approach ensures that quantitative changes detected are not due to the age of leaves, given that the density of leaf trichomes, where volatile production occurs, decreases as leaves age (Fischer et al., 2011). At weeks 2 and 3, seedlings were cut at the base of the hypocotyl with a razor blade, for fresh weight and height determination, and volatile collection. Ten to fifteen seedlings were used per replicate, with three biological replicates per experiment, and three independent experiments (October, February, and June). From weeks 4 to 6, 3 plants were used per replicate, with three replicates per experiment. The top leaf internode of individual plants was used for fresh weight measurement and volatile collection. To limit environmental variability measurements and collections were initiated at noon. Seedlings grown in enclosed chambers were collected at week 3.

### Volatile collection and analysis

For volatile collection, basil seedlings or leaves were harvested during two independent experiments, performed in October and February, with three biological replicates per experiment. Seedlings or leaves were collected as explained above and fresh weight was recorded. They were then loaded into thin walled glass tubes (2.5 cm i.d, 61 cm long and 300 ml volume) and connected to a push-pull dynamic headspace collection system. Filtered air was pushed into the headspace of each tube during 2 h so that the emitted volatiles were forced to pass into a glass column with a porous copolymer volatiles adsorbent trap (HaySep Q 80–100, Hayes Separations Inc., Bandera, TX). Methylene chloride was used to elute the volatiles from the trap and nonyl acetate was used as elution standard (Colquhoun et al., 2013; Johnson et al., 2016).

After elution, the samples were analyzed on a 7890B/5977A Series Gas Chromatograph/Mass Selective Detector (5977A MSD, Agilent Technologies, Santa Clara, CA, USA). The volatiles were separated in a DB-5 capillary column (5% phenyl 95% dimethylpolysiloxane, length: 30 m, ID: 0.250 mm, film thickness: 1 μm, Agilent Technologies, Santa Clara, CA, USA) using helium as carrier gas at 11.5 psi, temperature: 220°C. The initial oven temperature was 40°C (hold time: 0.5 min) and increased to 250°C (hold time: 4 min) at 5°C min^−1^ gradient rate. A post-run temperature of 260°C was applied. The transfer line, ion source and quadrupole temperatures were 280, 230, and 150°C, respectively. Extractor electronic impact (EI) ion source was set to 70 eV. The full scan mass range was 40–550 *m/z* (threshold: 150).

Volatile compound MS spectra were compared to NIST 11 database search for identification, and authentic standards (Sigma Aldrich, St Louis, MO, USA) for 44 compounds (of 62 total volatiles) were used to confirm identity by comparing the retention times. Where standards were not available compounds were identified based on results of previous analyses in different basil cultivars (Keita et al., 2000; Lewinsohn et al., 2000; Hiltunen and Holm, 2003; Sacchetti et al., 2004; Chalchat and Özcan, 2008; Gendo et al., 2008; Chang et al., 2009b), with the exception of four compounds. Table S1 lists the 62 compounds, and the individual species identified by NIST and confirmed with standards or found in the literature. Peaks from the chromatograph of compounds within a sample elution were automatically integrated using MassHunter Workstation Software Version B.06.00 software (Agilent Technologies, Inc., 2012). Relative peak areas of volatiles detected were adjusted to peak area of elution standard, biological sample mass seedling or leaves and time of collection (Colquhoun et al., 2013). Raw data can be found in Table S4.

### Measurement of total antioxidant capacity

Total antioxidant capacity was measured in 3 week-old greenhouse or controlled environment seedlings. Five to ten seedlings were used per replicate, with three biological replicates per experiment in three independent experiments (October, February, and June). Seedlings were cut at the basis of the hypocotyl, weight was recorded, and total material was used for antioxidant capacity analysis. In the greenhouse trial performed in June, only the top leaf internode, cut 1 cm below the internode, was used. The oxygen radical absorbance capacity-fluorescein (ORAC-FL) method was followed as previously established (Cao et al., 1993; Ou et al., 2001; Carvalho and Folta, 2014b). This method allows rapid and relatively inexpensive measurements of total antioxidant capacity. Crude extraction was performed in 80:19:1 (V/V) methanol/water/acetic acid solution. The extract was divided into three aliquots used as technical replicates within each independent experiment.

### Data analysis

Means of log-transformed weights and heights values were compared by One-way ANOVA and comparisons for each pair were evaluated by Student's *t*-test and Bonferroni correction. Leaf areas were compared by Kruskal-Wallis and multiple comparisons by Mann-Whitney-Wilcoxon test and Bonferroni correction were performed. Antioxidant capacity means were evaluated by Two-way ANOVA and comparisons for each pair were evaluated by Student's *t*-test and Bonferroni correction. These tests and calculation of means for total volatiles were determined using JMP (Version 12, SAS Institute Inc., Cary, NC, USA).

To build the developmental heat map, relative concentrations were normalized to a log scale and numbers compared for each individual during the five time points. To develop the heat map at 3 weeks in the greenhouse and controlled chambers, for each volatile compound median numbers were scaled and centered. Representative colors were obtained based on relative amount of each chemical across the seven conditions. Heat maps and univariate analysis of volatiles production were performed using R 3.2.3 (RDCTeam, 2015) and the packages coin (Hothorn et al., 2008) and *q*-value (http://qvalue.princeton.edu/, http://github.com/jdstorey/qvalue, (Storey, 2003) in RStudio (RStudioTeam, 2015). Medians of all volatile samples were compared by Mann-Whitney-Wilcoxon test and multiple testing hypothesis error evaluated by False Discovery Rate calculation (Vinaixa et al., 2012).

## Results

### Developmental pattern of basil flavor in the greenhouse

**Figure 1:**
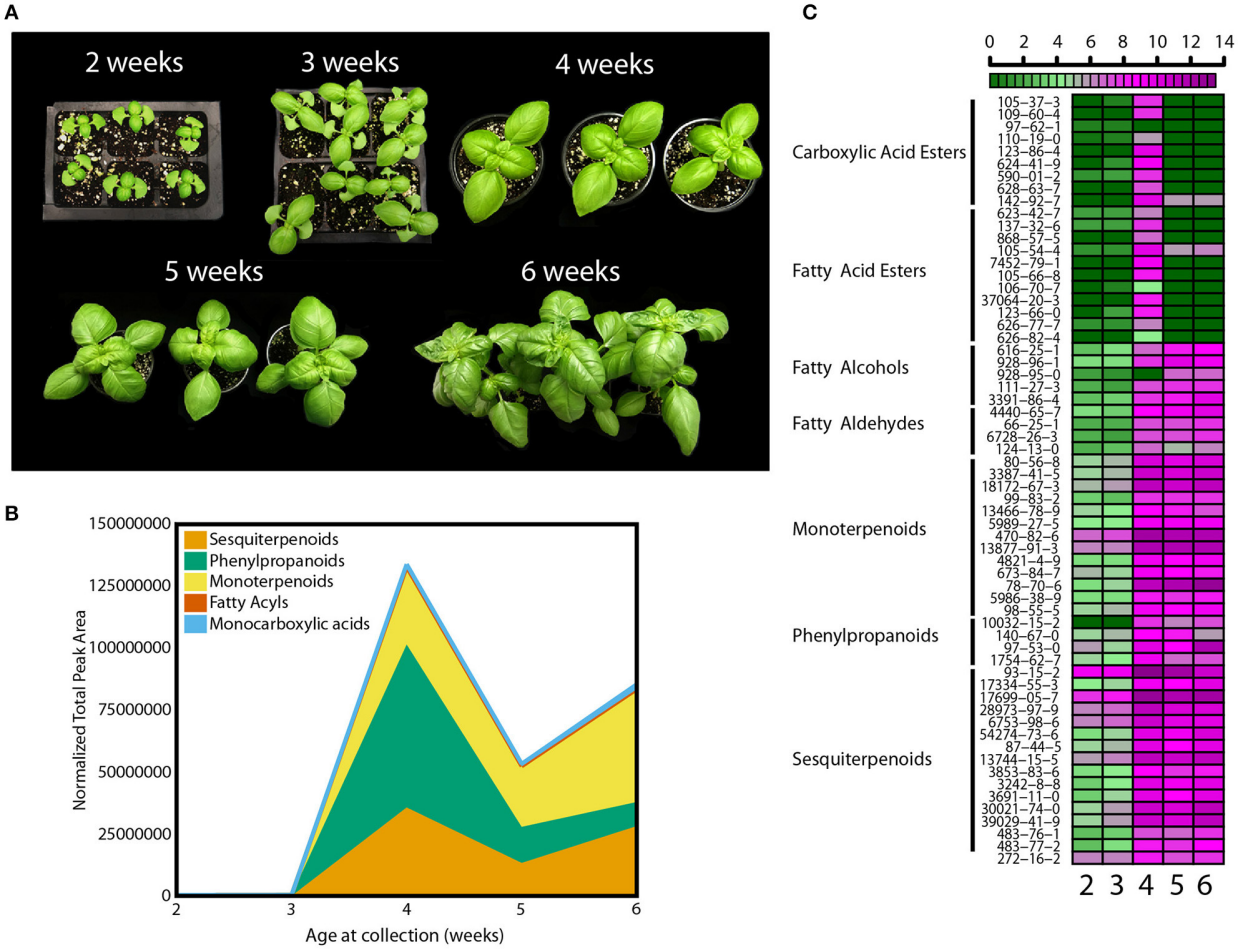
**Developmental effects on basil growth and metabolism. (A)** Representative pictures from 2 to 6 weeks of plants grown in the greenhouse during the fall with **(B)** quantification of total volatiles and means of 5 subclasses, normalized by total peak area, and **(C)** heat map for individual volatile species, with relative quantification within the 5 time points; green and purple indicate low and high levels, respectively, as shown in the scale bar. Results are representative of two independent experiments (*n* = 3).

The level of chemicals at the basis of basil flavor vary within plant tissues and developmental stages (Johnson et al., 1999; Ioannidis et al., 2002; Hakkim et al., 2007; Chang et al., 2009a). To standardize experimental conditions and plant material, a developmental assay was performed first. Basil seeds (*Ocimum basilicum* L. cv Caesar) were germinated in a glass-covered greenhouse and plants allowed to grow for 6 weeks. Beginning at 2 weeks after sowing, measures of plant development (morphological, metabolic, and physiological) were recorded. Every week leaf and stem growth along with the appearance of new leaf-pairs was measured (Figure 1A). Based on reported literature and empirical attempts, analytical methods were developed to collect, separate, identify, and relatively quantify 62 volatile molecules (Table S1) (Lee et al., 2005; Liber et al., 2011; Pirmoradi et al., 2013). These 62 volatiles can be classified into five major chemical classes: carboxylic acid esters (or monocarboxylic acids), fatty acyls (which include fatty acid esters, fatty alcohols and fatty aldehydes), monoterpenoids, phenylpropanoids, and sesquiterpenoids. Total volatile amounts increased between weeks 3 and 4 (Figure 1B). Relative amounts of volatiles within the five chemical classes also changed throughout the time course (see Table S2 for complete statistical analysis). At week 4, phenylpropanoids were found at the highest levels followed by sesquiterpenoids and monoterpenoids (*p* < 0.05, *q* < 0.1). This trend progressively changed thorough week 5, and by week 6 phenylpropanoids levels were at 15% of the levels measured at week 4 (*p* < 0.05, *q* < 0.1), while monoterpenoids showed a 1.5-fold change (*p* < 0.05, *q* < 0.1). Differences in more than 65% of sesquiterpenoids and monoterpenoids (*p* < 0.05 for each individual volatile, *q* < 0.001) were observed at week 6. A developmental heat map was constructed showing trends of individual volatile compounds within each class (Figure 1C). This diagram allows visualization of relative levels of individual compounds throughout the time course, going from dark green to dark purple to show lower and higher relative amounts, respectively. Amounts of most volatiles were low before week 4, and then rapidly increased. More than 85% of the compounds were detected at higher levels at this stage (*p* < 0.05 for each individual volatile, *q* < 0.001). All compounds were also affected by developmental cues. Monocarboxylic acids and fatty acyl volatiles peaked at week 4 (*p* < 0.05, *q* < 0.1), whereas the remaining classes maintained relatively higher levels after week 3. In terms of individual species, for example, the phenylpropanoid eugenol (CAS 97-53-0, Table S1) had two peaks at week 4 and 6 (*p* < 0.05, *q* < 0.001). Higher amounts were observed at week 6, despite the decreasing levels of phenylpropanoids as plants matured (Figure 1B). Another phenylpropanoid, estragole (CAS 140-67-0) peaked at week 4 and 5 (*p* < 0.05, *q* < 0.001). The monoterpenoid linalool (CAS 78-70-6) was detected at similar amounts for weeks 4 and 5 and peaked at week 6 (*p* < 0.05, *q* < 0.001). Other monoterpenoids such as 1,8-cineole (CAS 470-82-6) and two pinene compounds (CAS 80-56-8 and CAS 18172-67-3) had smaller variations and a significant increase at week 4 (*p* < 0.05, *q* < 0.01).

### Morphological differences induced by narrow-bandwidth light

**Figure 2:**
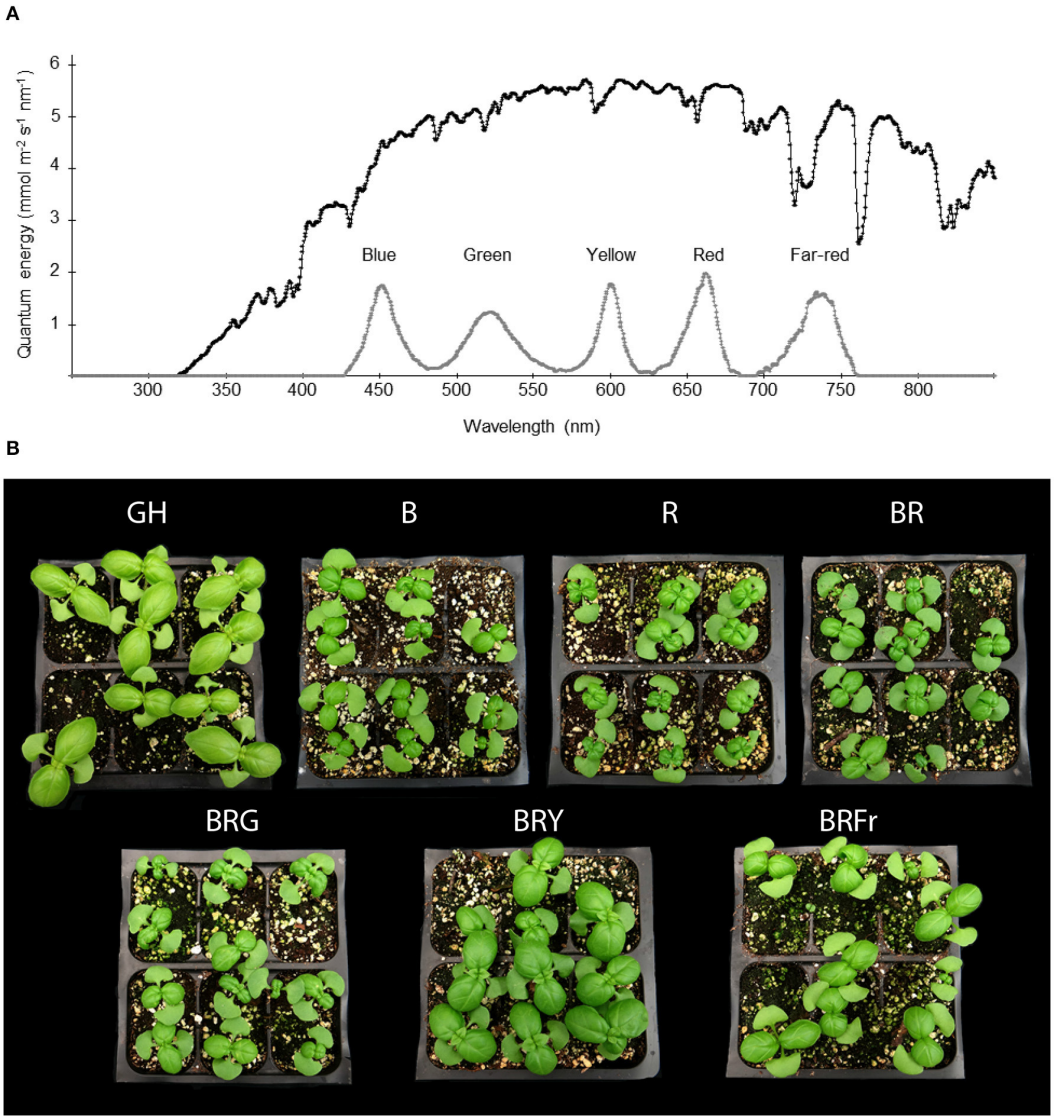
**Effects of light treatments on basil growth. (A)** Energy distribution of full sunlight in the greenhouse (black line) measured at 1 pm in July in Gainesville, Florida (29.6516°N) and in enclosed chambers irradiated with LED-based light sources (gray line), and **(B)** representative pictures of 3 week-old seedlings grown in October under greenhouse (GH) or narrow-bandwidth treatments; B, blue; R, red; G, green; Y, yellow; Fr, far-red.

Figure 1 describes the developmental pattern of the basil cultivar of “Ceasar” in a glass greenhouse. In this greenhouse, the solar energy distribution ranges from near UV to near-infrared wavelengths (Figure 2A). In order to ascertain the specific effects of narrow-bandwidth light on basil growth and development, closed chambers (temperature, humidity, and air flow controlled) exclusively illuminated with LED sources were employed. The light sources used for these experiments emit in blue (450 nm), green (520 nm), yellow/amber (600 nm; noted as “yellow” in the text), red (660 nm), and far-red (735 nm) wavebands (Figure 2A). These sources were used either individually or in combination. Basil seeds were germinated in enclosed chambers, and in the greenhouse for comparison, and allowed to grow for 3 weeks. Different light conditions were used, blue (B), red (R); B and R (BR, ratio 1:1), B, R and green (BRG, 1:1:1); B, R and yellow (BRY, 1:1:1), and B, R and far-red (BRFr, 1:1:1). Sweet basil plants germinated and grown under greenhouse and LED treatment conditions for 3 weeks displayed distinct phenotypes (Figure 2B). All LED treatments produced smaller plants compared to greenhouse conditions except the BRY treatment, which was similar to greenhouse grown sweet basil.

### Differences in volatile abundance under narrow-bandwidth light

**Figure 3:**
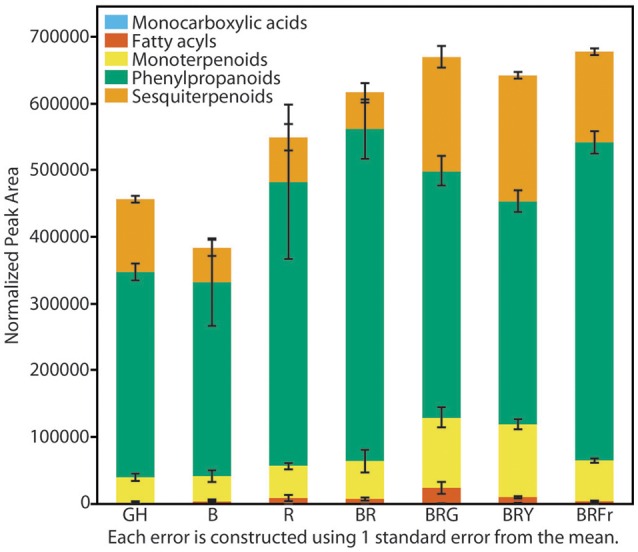
**Effects of light treatments on basil volatile profiles**. Quantification of total volatiles and 5 subclasses in 3 week-old seedlings grown in the greenhouse (GH) in October and six enclosed chambers irradiated with narrow-bandwidth treatments; B, blue; R, red; G, green; Y, yellow; Fr, far-red. Numbers are represented based on normalization of total peak area. Results are representative of two independent experiments (*n* = 3).

Total volatiles from 3- and 4-week-old sweet basil grown in the greenhouse and controlled environments were collected and analyzed. Phenylpropanoids were the most abundant species, followed by sesquiterpenoids and monoterpenoids (Figure 3). This pattern was generally observed under all light conditions (see Table S3 for detailed statistical analysis). B and BR showed lower relative accumulation of sesquiterpenoids compared to greenhouse treatment (*p* < 0.05, *q* = 0.083) while there was not statistical difference under R light. Relative amounts of major classes of volatiles remained consistent with greenhouse conditions, except for fatty acyls and carboxylic acid esters, which were more abundant in R, B and BR (*p* < 0.05, *q* = 0.083). Plants grown under BRG and BRY showed higher amounts of monoterpenoids, and decreased carboxylic acid esters and phenylpropanoids compared to BR (*p* < 0.05, *q* = 0.082), and showed the highest volatile levels when compared to greenhouse conditions. Green light treatment resulted in higher levels of fatty acyls. When far-red was added to BR, relative levels of sesquiterpenoids were higher compared to BR (*p* < 0.05, *q* = 0.083), whereas the remaining classes remained unchanged.

**Figure 4:**
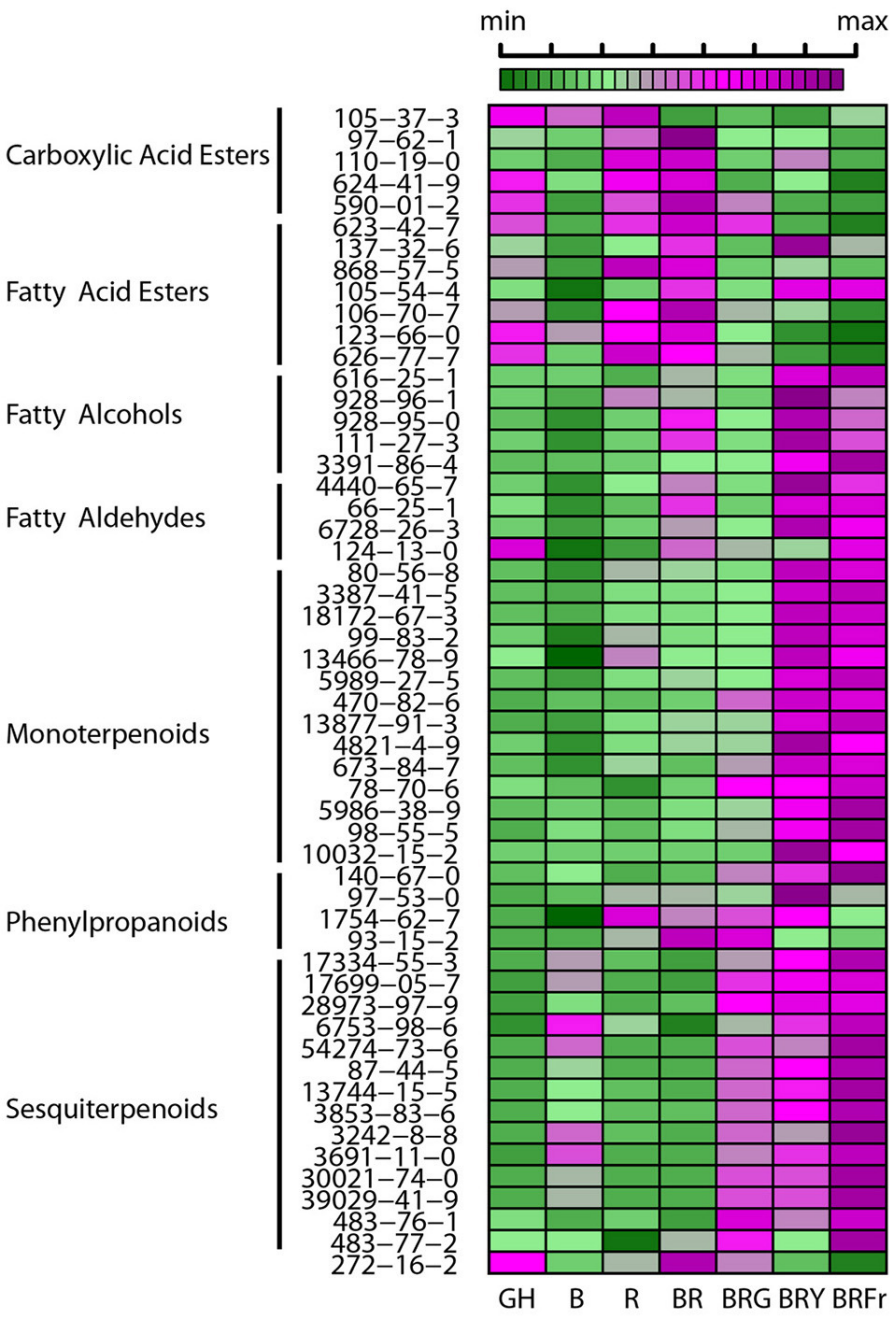
**Effects of light treatments on basil individual volatile species**. Heat map showing relative levels of basil volatile compounds in 3 week-old seedlings grown in the greenhouse (GH) in October and six enclosed chambers with artificial lighting; B, blue; R, red; G, green; Y, yellow; Fr, far-red. CAS numbers area shown, for chemical names see Table S1. For each compound medians were scaled and centered, and color-codes built based on comparison thought the seven conditions. Green and purple indicate low and high levels, respectively, as shown in the scale bar. Results are representative of two independent experiments (*n* = 3).

A heat map was constructed to visualize the effects of the various light conditions on individual volatile molecule types and broad chemical classes. All volatile molecules were responsive to differences in the light environment (Figure 4). Statistical comparisons of the two experimental replicates, in October and February, revealed that some volatile compounds were consistently and significantly different between light treatments. Tables 1, 2 list these individual compounds that consistently differentially accumulated between light treatments. BR treatment produced plants similar to the greenhouse, except for two sesquiterpenoids (α-humulene, and α-bulnesene) that were found at higher levels in October in greenhouse conditions. Adding a third wavelength to BR resulted in plants with distinctive volatile compositions. Green added to BR consistently promoted accumulation of sesquiterpenoids and monoterpenoids, as well as the phenylpropanoids eugenol and estragole. 1,8-cineole was among the monoterpenoids induced by G supplementation. BRG compared to GH also showed significant higher accumulation of linalool. Yellow added to BR consistently induced sesquiterpenoids and monoterpenoids, together with the phenylpropanoid estragole. Eugenol, 3-hexenal, (Z)-3-hexen-1-ol, and hexanol were the only compounds consistently and significantly different between BRY and BRG, with these volatiles being decreased in BRY treatments. Conversely, far-red addition to BR consistently induced the sesquiterpenoid but not the monoterpenoid class of volatiles. Compared to GH, BRFr also promoted accumulation of a few volatile compounds from other chemical classes such as 1,8-cineole and the fatty acyl 1-octen-3-ol.

**Table 1:** **Volatile compounds from 3 week-old basil seedlings that increase in abundance between light treatments (fold-change > 1.5, ***p*** < 0.05, ***q*** < 0.01)**.

**B vs. GH**	**RBG vs. GH**	**RBY vs. GH**	**RBFr vs. GH**	**RBG vs. RB**	**RBY vs. RB**	**RBFr vs. RB**	**RBFr vs. RBY**
Octanal	(Z)-beta-farnesene	alpha-pinene	(Z)-beta-farnesene	(+)-calarene	beta-caryophyllene	(Z)-beta-farnesene	Methyl (E)-cinnamate
	beta-caryophyllene	sabinene	beta-cubebene	alpha-bergamotene	gamma-muurolene	beta-caryophyllene	
	beta-cubebene	laevo-beta-pinene	alpha-himachalene	(Z)-beta-farnesene	Estragole	gamma-muurolene	
	alpha-himachalene	dextro-limonene	gamma-muurolene	alpha-humulene	laevo-beta-pinene	(+)-calarene	
	gamma-muurolene	1,8-cineole	(R)-gamma-cadinene	1-Epi-bicyclosesquiphellandrene	beta-ocimene	alpha-humulene	
	Estragole		alpha-phellandrene	beta-caryophyllene	alpha-humulene	beta-cubebene	
	Eugenol		1,8-cineole	beta-cubebene	beta-cubebene	(R)-gamma-cadinene	
	alpha-pinene		alpha-terpineol	alpha-himachalene	(R)-gamma-cadinene	alpha-bergamotene	
	sabinene		1-octen-3-ol	Estragole	alpha-pinene	1-Epi-bicyclosesquiphellandrene	
	laevo-beta-pinene		4-terpinenyl acetate	Eugenol	alpha-phellandrene	alpha-himachalene	
	alpha-phellandrene			1,8-cineole	1-Epi-bicyclosesquiphellandrene	alpha-bulnesene	
	dextro-limonene			alpha-bulnesene	alpha-himachalene	Estragole	
	Ocimenol			gamma-muurolene	alpha-bulnesene	4-terpinenyl acetate	
	alpha-terpineol			(R)-gamma-cadinene	sabinene		
	1-Penten-3-ol			alloocimene	dextro-limonene		
	3-Hexenal			Ocimenol			
	Hexanal			alpha-terpineol			
	(Z)-3-hexen-1-ol			3-Hexenal			
	1-octen-3-ol			4-terpinenyl acetate			
	linalool						
	beta-ocimene						
	alloocimene						
	1,8-cineole						
	4-terpinenyl acetate						

*GH, greenhouse; B, blue; R, red; G, green; Y, yellow; Fr, far-red*.

**Table 2:** **Volatile compounds from 3 week-old basil seedlings that decrease in abundance between light treatments (fold-change < 0.67, ***p*** < 0.05, ***q*** < 0.01)**.

**R vs. GH**	**B vs. GH**	**RB vs. GH**	**RBY vs. RBG**	**RBFr vs. RBG**
(+)-calarene	Estragole	alpha-humulene	Eugenol	Ocimenol
alpha-bergamotene		alpha-bulnesene	3-Hexenal	1-Penten-3-ol
(Z)-beta-farnesene			(Z)-3-hexen-1-ol	3-Hexenal
1-Epi-bicyclosesquiphellandrene			Hexanol	Hexanal
beta-caryophyllene				(Z)-3-hexen-1-ol
alpha-terpineol				Hexanol
1-octen-3-ol				

*GH, greenhouse; B, blue; R, red; G, green; Y, yellow; Fr, far-red*.

### Physiological responses of basil to narrow-bandwidth light

**Figure 5:**
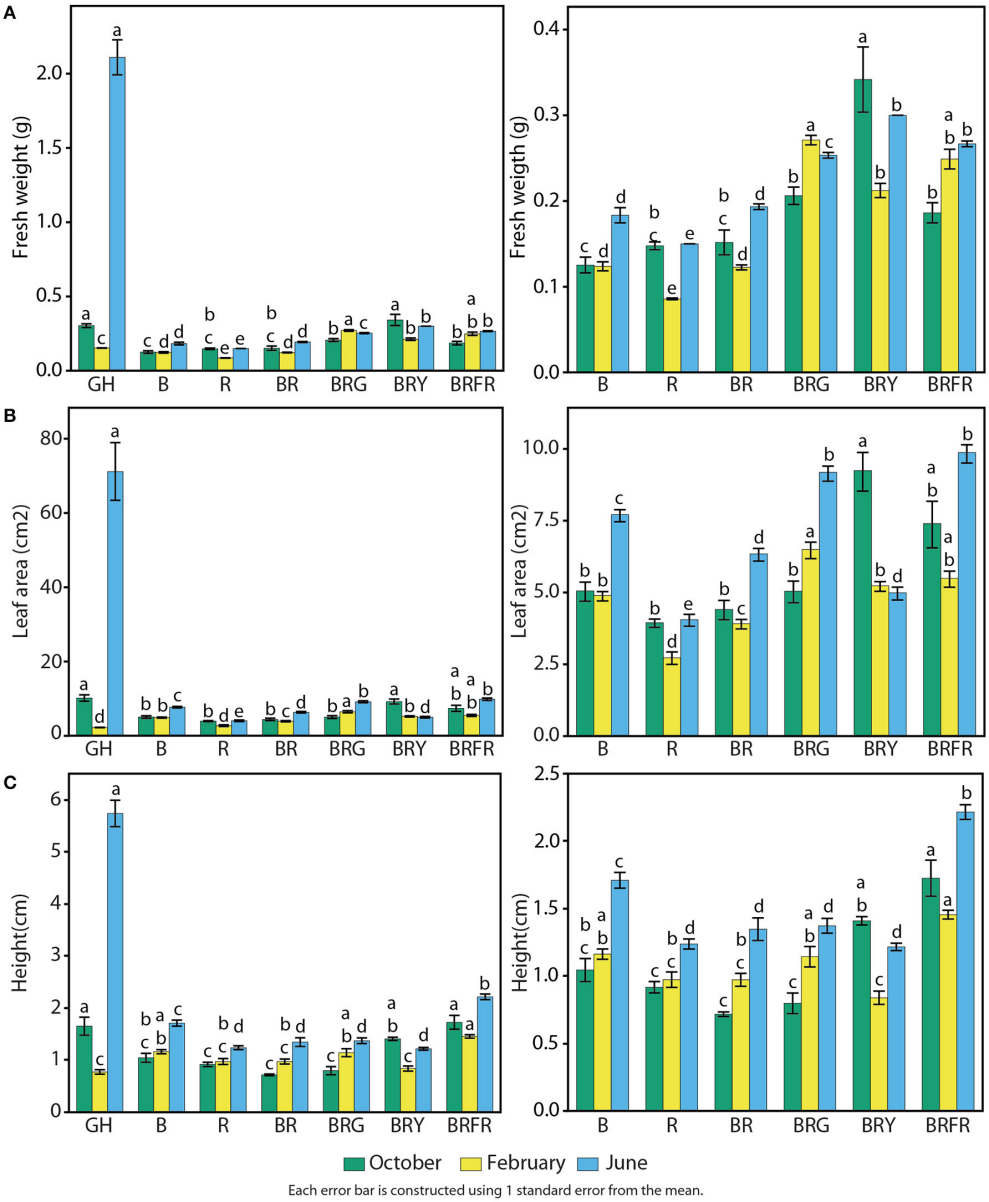
**Effects of environmental cues on physiological parameters of sweet basil**. Quantification of basil growth under greenhouse (GH), left panels, and artificial light conditions, left and right panels; right panels show an enlargement of panels on the left without greenhouse conditions; B, blue; R, red; G, green; Y, yellow; Fr, far-red; for 3 weeks during three different seasons, in terms of **(A)** weight, **(B)** total leaf area, and **(C)** height per/of seedling; green bars represent October, yellow February, and blue June; error bars represent standard error, *n* = 3; different letters indicate different values within the same season (*p* < 0.01).

Growth and physiological characteristics of sweet basil in the greenhouse and under the different narrow-bandwidth treatments were measured. Seasonal effects were taken into account by performing experiments during three different seasons of the year: fall, winter, and summer. At 3 weeks, seedling fresh weight, height, and total leaf area were measured (Figure 5). Seasonal differences resulted in a considerable variation of basil biomass in the greenhouse (Figure 5A, left panel). Higher and lower fresh weights were observed during the summer and winter, respectively. During the summer, greenhouse grown plants weighed 10 times more than under artificial lighting (2.0 g vs. an average of 0.2 g). This difference was not observed during the other two seasons. In the fall, the fresh weight of BRY-grown plants was similar to plants in the greenhouse, and during the winter all artificial light treatments promoted higher weight than greenhouse conditions (*p* < 0.003 per each comparison with GH). Controlled environment conditions produced basil plants with overall constant weight during the three seasonal replicates. B and R generated 0.15 g of fresh weight per seedling, which was equal to plants grown under B and R (Figure 5A, right panel). A third light quality added to BR led to increased weight compared to BR (*p* < 0.003). Higher values were measured under BRG (0.25 g) and BRY (0.30 g).

Similar effects of light conditions and seasons were observed on total leaf area (Figure 5B). Compared to artificial light conditions, leaf area in the greenhouse was higher during the summer (70 cm^2^), similar in the fall (10 cm^2^), and lower during the winter (Figure 5B, left panel). Total leaf area was generally constant in each artificial light treatment throughout the year. B generated larger leaves than R (5 vs. 3.5 cm^2^), and combination of both resulted in intermediate levels of total leaf area (Figure 5B, right panel). Adding a third waveband promoted leaf expansion when compared to BR (*p* < 0.003).

Basil height was greatest during the summer in the greenhouse (6 cm), comparable to artificial light conditions during the fall (1.5 cm), and slightly lower (0.8 cm) than most controlled conditions in the winter (Figure 5C, left panel). All light conditions containing R resulted in lower height than B alone (Figure 5C, right panel). The only exception was when Fr was present, which resulted in the highest measured values (1.8 cm on average).

### Biochemical responses of basil to narrow-bandwidth light

**Figure 6:**
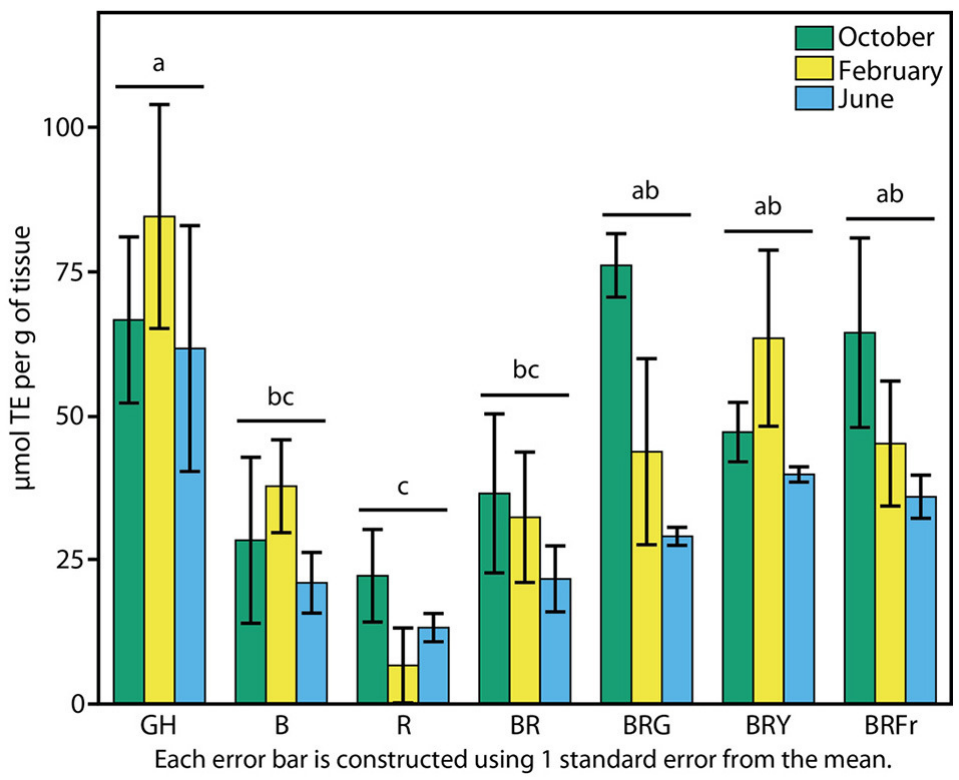
**Effects of environmental cues on biochemical properties of sweet basil**. Effects of light on antioxidant capacity of basil grown for 3 weeks under greenhouse (GH) and LED chambers; B, blue; R, red; G, green; Y, yellow; Fr, far-red. Total antioxidant activity measured in October, February, and June (green, yellow and blue bars, respectively); error bars represent standard error, *n* = 3. Different connecting letters indicate statistically significant differences between treatment group means (*p* < 0.015).

The reported antioxidant properties of basil prompted a test of total antioxidant activity. Sweet basil seedlings grown in the greenhouse and under narrow-bandwidth conditions were compared. The ORAC-FL assay was used to determine concentrations of Trolox (vitamin C analog) equivalents (TEs) of 3-week-old seedlings (Cao et al., 1993; Ou et al., 2001). Experiments were performed during three seasons as described above. Contrary to what was observed in terms of physiological responses, seasonal effects were less evident over TE accumulation in the greenhouse, and there was not significant statistical interaction between the season and the light treatments (*p* > 0.5). Fall, winter, and summer grown greenhouse plants displayed similar levels of antioxidant capacity: 60–80 μmol TE per unit seedling weight (Figure 6). Antioxidant levels were lowest under R, and three to five times lower than in the greenhouse (*p* < 0.003). B treatments resulted in slight increases, comparable to BR (30 μmol TE g^−1^). An induction in total antioxidant capacity to 40 μmol TE g^−1^ occurred when a third waveband was added to BR (*p* < 0.003), and no statistical differences were found between these conditions and the greenhouse.

### Persistence of the phenotypes

Basil seedling growth characteristics were measured under various light treatments for 3 weeks. The treatments resulted in altered basil metabolite profiles and presumably compounds associated with sensory quality. To examine the persistence of this effect after removal of specific light cues, 3-week-old seedlings were transferred from controlled environment conditions to greenhouse conditions along with appropriate controls, and volatiles were collected and analyzed 1 and 2 weeks later (weeks 4 and 5). These tests were performed in October and February and showed major differences.

**Figure 7:**
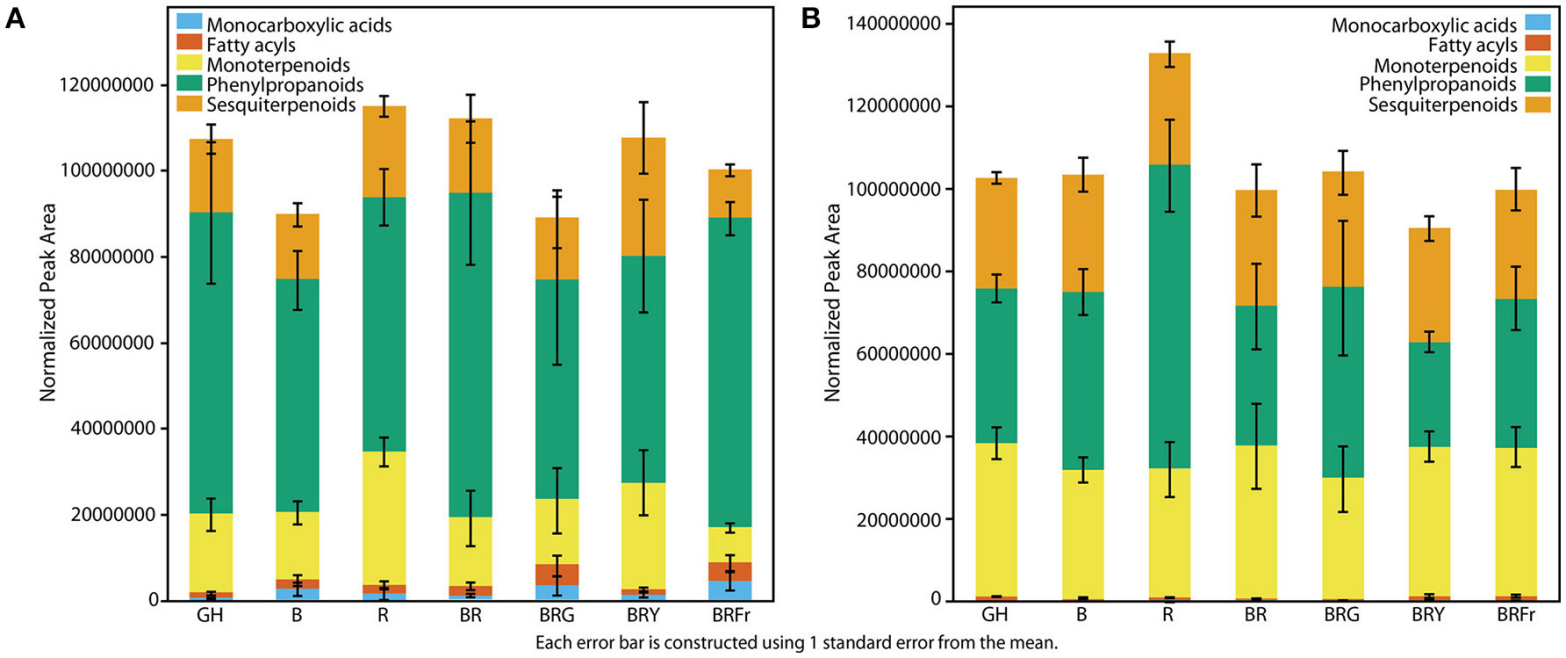
**Persistence of specific light treatments on basil flavor compounds**. Quantification of total volatiles and 5 subclasses in **(A)** 4 week-old, and **(B)** 5 week-old basil plants removed at week 3 from greenhouse (GH) and six narrow-bandwidth treatments; B, blue; R, red; G, green; Y, yellow; Fr, far-red; and transferred to the greenhouse. Numbers are represented based on normalization of total peak area. Results represent an experiment performed in October (*n* = 3).

Consistent with the developmental series (Figure 1B) volatile compound levels in greenhouse grown plants were higher at week 4 (Figure 7A) than week 3 (Figure 4) at the October harvest. The trend observed at week 3 in terms of volatile modulation by specific light cues was maintained at week 4 for the majority of the conditions. Sesquiterpenoids were lower in B or R compared to the greenhouse. The differences between greenhouse and controlled environment conditions from weeks 3 to 4 are that B and R resulted in a relatively lower amount of monoterpenoids, while fatty acyls and carboxylic acids slightly increased. The induction of relative levels of monoterpenoids by BRG and BRY observed at week 3 were lower at week 4. The inductive effect of BRFr on sesquiterpenoid accumulation also was no longer observed.

At week 5 plants grown in all conditions produced similar volatile levels and relative profiles (Figure 7B). Under these conditions the sweet basil plants were allowed to grow until the appearance of first flower buds to test for any effect of the light treatments on flowering time. No relevant differences were observed between any controlled environment and the greenhouse (data not shown).

## Discussion

Sweet basil (*Ocimum basilicum*) is highly appreciated as a food product, being used fresh, dried, or processed. Yield and aroma composition are fundamental for basil growers, and the basis for its sensory qualities is well established. *Ocimum* species are polyploid, and basil's delicate flavors result from complex combinations between dozens of chemical compounds (Liber et al., 2011; Pirmoradi et al., 2013). Constant demand for new flavors led to numerous breeding approaches that have created many varieties with distinct chemical compositions. Analysis by gas chromatography/mass spectrometry (GC/MS) combined with cluster analysis has allowed identifying important chemical components and classifying these chemotypes. Main constituents are terpenoids and phenylpropanoids. The monoterpenes linalool and 1,8-cineole, the sesquiterpenes α-bergamotene and α-farnesene, and the phenylpropenes eugenol, estragol, (*Z*)-methyl cinnamate, methyl eugenol, and isoeugenol are usually the more abundant. Volatile abundance is also highly dependent on the tissue tested, the age of the plant, season, and growing site (Hakkim et al., 2007; Hussain et al., 2008; Al-Kateb and Mottram, 2014; Sims et al., 2014). Synthesis and storage of volatiles occurs in leaf surface peltate glands, and enzymatic activity in different pathways correlates with presence of specific volatiles in different basil cultivars (Gang et al., 2001; Iijima et al., 2004a,b). It has been suggested that volatile compounds may have potential human health benefits, such as antioxidant, anti-allergic, antimicrobial, antifungal, anti-proliferative, or immuno-stimulatory properties (Lee et al., 2005; Bayala et al., 2014).

While the majority of efforts have sought to perfect aroma and flavor using genetics and breeding, some reports have demonstrated that aromatic compounds may be modulated by influences form the light environment (Loughrin and Kasperbauer, 2003). This report examines the action of different light treatments on the development, physiology, metabolism, and biochemistry of sweet basil seedlings, demonstrating effects on plant size and stature, but also the relative effects on changing volatile profiles that might affect flavors and aromas. These results may inform lighting strategies for crop producers to add value to a given product.

Basil seedlings demonstrated significant plasticity in volatile accumulation based on the ambient light environment. Volatile profiles of seedlings grown under BRG, BRY, and BRFr were consistently different from the greenhouse conditions during the two seasons tested. Addition of green or yellow to a background of BR induced accumulation of monoterpenoids and decreased phenylpropanoids. Far-red light only induced sesquiterpenoids. Other classes were generally not affected, with some exceptions in individual volatile molecules. Green light has been described in the past to affect the phenylpropanoid pathway in lettuce (Johkan et al., 2012). In basil, effects of visible wavelengths on volatile content have only been described using reflective colored plastic mulches. Significantly higher levels of aroma compounds and phenolic compounds have been measured in basil plants grown over green and yellow mulches (Loughrin and Kasperbauer, 2001, 2003). Colored plastic mulches have also changed aromas of strawberry fruits (Kasperbauer et al., 2001). Few reports have addressed effects of visible light on flavor of plant products. Approaches with narrow-bandwidth LED irradiation have only used short exposure times. Blue and red applied for 3 days pre-harvest modified volatile content of tea leaves, when compared to dark and natural light treatments (Fu et al., 2015). Both treatments induced volatile fatty acid derivatives, phenylpropanoids, and terpenes. Molecular analyses revealed light activation of genes involved in volatile synthesis, such as *9/13-lypoxygenases* (fatty acids pathway), *phenylalanine ammonialyase* (phenylpropanoids), and *terpene synthases*. Petunia flowers exposed for 8 h to red and far-red light resulted in altered volatile benzenoid/phenylpropanoid emission, and levels of the key floral volatile 2-phenylethanol were increased when compared to white, blue or dark treatments (Colquhoun et al., 2013). Strawberry, tomato, and blueberry were also targeted at post-harvest for 8 h, and their volatile profiles were modified with light.

In this report light treatments were extended for longer periods. A broader comprehensive approach and metabolomics analyses specify the effects of narrow-bandwidth lights on secondary metabolism and aroma-compound formation in plants. Green/yellow and far-red appear to cause different effects. Genes responsible for floral compound synthesis in petunia are regulated in similar manner, and emissions depend on substrate availability (Colquhoun et al., 2010; Spitzer-Rimon et al., 2010). Future analyses may use photosensory mutants to help pinpoint mechanisms linking light signaling and volatile formation. Putative green and/or yellow receptors are however yet to be found (Folta and Maruhnich, 2007). It will also be interesting to combine green and yellow and test synergistic actions on volatile modulation.

BRG, BRY, and BRFr treatments also altered profiles of many individual compounds known to affect sensory quality and consumer preferences. The heat maps we constructed are powerful tools to look at behavior of these species. Compared to greenhouse conditions, eugenol was induced by BRG. Eugenol has a strong clove-related flavor, and is used in many industries, from cosmetics, to food applications as a flavoring agent, or medicine. It has been described as antioxidant, antimutagenic, antigenotoxic, anti-inflammatory, and potentially possessing anticancer properties (Prakash and Gupta, 2005; Jaganathan and Supriyanto, 2012). BRG also promoted higher levels of linalool, which is at the basis of sweeter/floral aromas. This compound also has been described to possess antioxidant, anti-inflammatory, and neuroprotective effects and has also been used to combat rice pests (Lopez et al., 2008; Park et al., 2016). BRG, BRY, and BRFr promoted accumulation of 1,8-cineole, a compound with a spicy and camphor-like aroma, and potentially important therapeutic properties (Ryu et al., 2014). Slight alterations in less abundant species can possibly have significant impacts on flavors. Human sensory panels may be considered in the future to test this hypothesis. It is exciting to speculate that specific light programs may be designed to create flavors targeting personal preferences and healthier food products. This outcome could also be achieved by decreasing levels of toxic compounds, such as methyl eugenol (De Vincenzi et al., 2000).

Extensions of this research may apply these light combinations to other aromatic herbs. In some aromatic herb crops UV has been used for modulation of aroma. Examples of induction of flavor-related compounds by UV have been reported in basil and peppermint (Johnson et al., 1999; Chang et al., 2009a; Behn et al., 2010; Dolzhenko et al., 2010; Hikosaka et al., 2010; Bertoli et al., 2013). These wavebands have even been suggested to be required for normal development of oil glands in basil (Ioannidis et al., 2002). These treatments may be synergistic to the effects of visible light outlined in this work. UV light triggers stress responses that induce synthesis of protective pigments, and would also perhaps positively affect antioxidant capacity (Sakalauskaitė et al., 2013). It has also been shown that supplemental red light increases antioxidant levels in parsley and dill (Bliznikas et al., 2012).

Greenhouse-grown basil plants were highly affected by seasonal effects which brought changes in photoperiod, spectrum and temperature. Producers that want reliable and regular year-round production cycles may employ a combination of BRG, BRY or BRFr (1:1:1, 150 μmol m^−2^ s^−1^) in enclosed chambers, as higher weight and leaf areas were consistently seen over the year. It is also likely that supplemental lighting could compensate for the seasonal changes.

Ratios of far-red to red light can also impact growth, development and stress tolerance, and therefore yield and quality, so future trials will explore ratios beyond the 1:1 RFr that was used in this work. Additional consideration must be given to individual species and developmental specific responses (Demotes-Mainard et al., 2016). In basil, special attention should be given at maintaining relative high levels of red light. This region of the spectra inhibits *Peronospora belbahrii* oomycete sporulation, the basis for downy mildew disease, which is of great concern in basil horticulture (Cohen et al., 2013).

Growth under narrow-bandwidth B resulted in seedlings similar to BR with some enhancement in leaf area. Many studies have examined blue combined with red as light source for growth of crops in controlled conditions. Species and cultivar specific responses are crucial. Different ratios and intensity have been tested in basil, cucumber, tomato, rapeseed, or lettuce, and proven optimization to be complex (Tarakanov et al., 2012; Fan et al., 2013; Li et al., 2013; Lin et al., 2013; Hernandez and Kubota, 2016). In cucumber a BR ratio of 1:1 allows optimum leaf development, maximum photosynthesis, and chlorophyll contents (Hogewoning et al., 2010). In this work BR (1:1) did not result in what might be considered most horticulturally optimal basil growth when compared to any combination of three wavelengths.

Light spectra and intensity, temperature, and photoperiod, are crucial parameters that remained static in the controlled environment experiments. Plants in the greenhouse were quantitatively similar to these artificial conditions during the fall, but had better overall growth characteristics in summer, and were generally worse in winter. Controlled environment chambers were set at constant 24°C. In Gainesville, North Florida, averages daily/night temperatures during those periods are 27°C/13°C, 33°C/22°C, and 21°C/7°C, respectively (www.usclimatedata.com). The greenhouse grown plants experienced relatively high sunshine, and photoperiod ranged from approximately 11–14 h 30 min. The improved relative growth observed in the summer in the greenhouse suggests it may be useful to increase temperature in the controlled environments. These attempts must also consider if beneficial effects seen by specific light signals on volatile profiles are maintained at higher temperatures.

Typically, plants quickly adjust to the light environment to optimize growth, so it was important to assess how long the induced changes persisted. Volatile profiles were generally maintained for approximately 1 week after treatment, while the effects were not observed at 2 weeks. These observations suggest that treatments may augment sensory quality for 2 weeks, framing a timeline for distribution and consumption of an optimal product. Temperature should be maintained at levels comparable to the growth chambers during this period, based on the observation that more variability was observed during the winter persistence experiment compared to fall (data not shown).

The volatile compound retention results also suggest that specific light treatments during germination and early seedling development may install a particular developmental/metabolic pattern that influences the potential to produce flavor and aroma compounds later. After reaching a certain developmental threshold plants delay adjustment to new conditions. This hypothesis would imply increased difficulty to modify sensory characteristics in mature plants, once they have undergone early developmental programming. This hypothesis was tested by measuring volatile profiles at other stages of development, with no favorable outcomes (data not shown).

These trials demonstrate that seedling growth and development may be modulated with changes in the light environment, and suggests that narrow-bandwidth light treatments might be a replacement for, or supplement to, ambient solar radiation. The changes in volatile profiles and morphology suggest that tailoring of light conditions may increase the value and quality of high-value herbs grown for human sensory characteristics.

## Author contributions

SC and MS performed the trials, propagated plants, performed the measurements. MS and CA performed volatile assessments and statistical treatments therein. SC and CA compiled the data and performed statistical analysis. SC, TC, and KF participated in preparation of the manuscript. SC, TC, and KF provided scientific oversight in experimental design and interpretations. TC and KF obtained funding for the study.

## Funding

This work was supported by the United States Department of Agriculture's National Institute of Food and Agriculture (USDA-NIFA, Award 2015-67017-23078), Florida Department of Agriculture and Consumer Services (USDA-FDACS), and the UF/IFAS Plant Innovation Center. All of KF's research and outreach funding, as well as travel reimbursements are available at www.arabidopsisthaliana.com/funding.html.

### Conflict of interest statement

The authors declare that the research was conducted in the absence of any commercial or financial relationships that could be construed as a potential conflict of interest. The reviewer MF-F and handling Editor declared their shared affiliation, and the handling Editor states that the process nevertheless met the standards of a fair and objective review.
